# Light-driven molecular switch for reconfigurable spin filters

**DOI:** 10.1038/s41467-019-10423-6

**Published:** 2019-06-05

**Authors:** Masayuki Suda, Yuranan Thathong, Vinich Promarak, Hirotaka Kojima, Masakazu Nakamura, Takafumi Shiraogawa, Masahiro Ehara, Hiroshi M. Yamamoto

**Affiliations:** 1 Institute for Molecular Science, Myodaiji, Okazaki, 444-8585 Japan; 2 RIKEN, Wako, Saitama, 351-0198 Japan; 3 SOKENDAI (Graduate University for Advanced Studies), Myodaiji, Okazaki, 444-8585 Japan; 4 School of Chemistry, Institute of Science, Suranaree University of Technology, Nakhon, Ratchasima 30000 Thailand; 5 School of Molecular Science and Engineering, Vidyasirimedhi Institute of Science and Technology (VISTEC), Rayong, 21210 Thailand; 6 Division of Materials Science, Nara Institute of Science and Technology, Nara, 630-0192 Japan

**Keywords:** Molecular machines and motors, Electronic and spintronic devices

## Abstract

Artificial molecular switches and machines that enable the directional movements of molecular components by external stimuli have undergone rapid advances over the past several decades. Particularly, overcrowded alkene-based artificial molecular motors are highly attractive from the viewpoint of chirality switching during rotational steps. However, the integration of these molecular switches into solid-state devices is still challenging. Herein, we present an example of a solid-state spin-filtering device that can switch the spin polarization direction by light irradiation or thermal treatment. This device utilizes the chirality inversion of molecular motors as a light-driven reconfigurable spin filter owing to the chiral-induced spin selectivity effect. Through this device, we found that the flexibility at the molecular scale is essential for the electrodes in solid-state devices using molecular machines. The present results are beneficial to the development of solid-state functionalities emerging from nanosized motions of molecular switches.

## Abstract (web summary)

The chirality provides new route for organic materials to be implemented in the spintronics applications. Here the authors show a solid-state spin-filtering device in an organic spin-valve structure enabled by light irradiation induced change in the chirality of molecule.

## Introduction

In spintronics, the use of organic materials as a spin transport material has recently garnered significant attention as they exhibit long spin-relaxation times and long spin-diffusion lengths owing to the weak spin–orbit interaction (SOI) of light elements^1–7^. Meanwhile, the weak SOI of organic materials become a drawback when they are used as a spin filter (or spin polarizer), In addition, the exchange interaction in an organic material is extremely weak to induce a ferromagnetic transition above room temperature. A spin-polarized current is, therefore, typically generated by inorganic materials with ferromagnetism or strong SOIs^8–13^. However, the recent finding of spin-selective electron transport through chiral molecules by Naaman et al., i.e., the so-called chirality-induced spin selectivity (CISS) effect, suggests an alternative method of using organic materials as spin filters for spintronics applications^14–17^. Although its microscopic mechanism is still under debate, the CISS effect has been validated by numerous experiments such as photoelectron spectroscopy^14,18^, magnetoresistance (MR) measurements, photoinduced electron transfer measurements^19–21^, spin-polarized conductive atomic force microscopy (AFM)^22–24^, single-molecule scanning tunnel microscopy^25^, and X-ray circular dichromism measurements^26^. Moreover, the CISS effect was already demonstrated in several applications such as magnetic memory devices^27–29^, spin-injection devices^30^, water splitting^31,32^, and enantiomer separation^33,34^. However, in all of the cases above, chiral molecules used in the experiments are static molecules such as DNAs^14,19,22,33^, helicenes^18,23^, amino acids^35,36^, and peptides^20,21,25,26,28,29,37,38^. Hence, the manipulation of spin-polarization (SP) direction by external stimuli has not been realized yet.

**Fig. 1:**
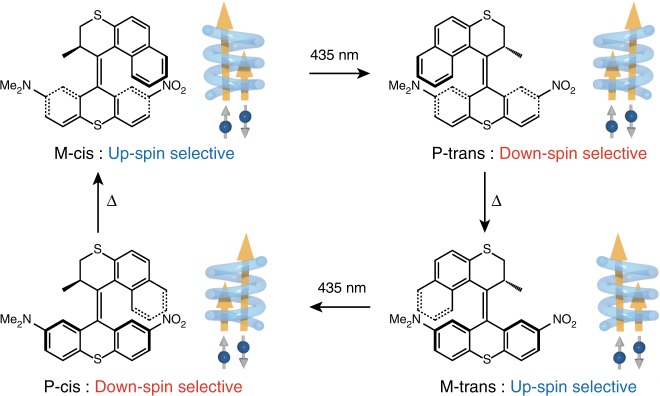
Molecular structures of OCA. Unidirectional rotation of the OCAs. The rotation cycle includes 4 times chirality inversion, resulting in 4 times switching of the spin-polarization direction of currents

In this context, overcrowded alkenes (OCAs) that demonstrate molecular motor functionalities are promising for integration in CISS-based SP solid-state devices because they demonstrate chirality switching by external stimuli. The first unidirectional molecular motor was reported in 1999 by Feringa et al.^39^. A cis-trans photoisomerization around the central C–C double bond is a basic driving force of the molecular rotation. Owing to the asymmetric stereo chemistry around the double bond, the system undergoes a unidirectional 360° rotation (Fig. 1a). The rotation cycle includes four distinct steps: two photochemical and two thermal steps, resulting in 4 times P/M chirality inversion during the 360° molecular rotation. Because the SP in the CISS effect depends on the chirality of the molecules, namely P (right handed) or M (left handed), the SP direction of electrons that pass through the molecular motors should be switched by light irradiation or thermal treatments^40–42^.

## Rusults

### MR measurements with gold top electrodes

**Fig. 2:**
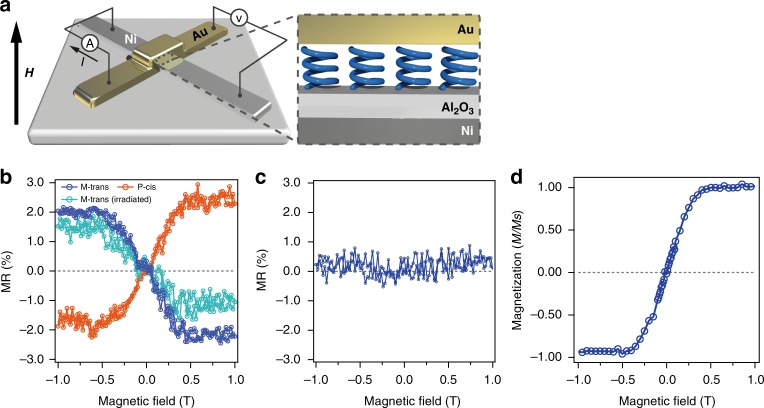
MR measurements with Au top electrode. **a** Schematics of the Ni(50 nm)-Al_2_O_3_(3 nm)-OCAs(2–3 nm)-Au(20 nm) cross-bar tunnel junction device. Blue helices represents the OCAs (the actual molecular orientation might be disordered). **b** MR curves for the devices fabricated individually with M-trans isomer (blue plot), M-trans isomer after irradiation with visible light for 5 min (sky blue plot), and P-cis isomer (orange plot). The percentage of MR was calculated from the in-field-measured resistance, *R(H)*, and the zero-field resistance *R(H* *=* *0)* as MR(%) = 100 × *[R (H) − R (H* *=* *0)]/ R (H* *=* *0))*. Sky blue plots show MR after photoirradiation. **c** MR curve for the device fabricated with racemic OCAs before optical separation. **d** Magnetization curves for Ni/Al_2_O_3_ electrode

To confirm spin-polarized current generation by an OCA molecule, a solid-state device that sandwiches a thin layer of an OCA molecule with Al_2_O_3_ (3 nm)/Ni (50 nm) and a gold (20 nm) electrode (Fig. 2a) was fabricated. The OCA film was prepared by the spin-coating method and a random molecular arrangement is expected (AFM images for the OCA films are shown in Fig. S7). However, we emphasize that finite spin-polarization owing to CISS effects can be expected even for a completely random orientation because the molecular chirality cannot be canceled out. External magnetic fields of upto 1 T were applied perpendicular to the substrates during the measurement, where the magnetization direction of the Ni electrode can be switched between the up and down directions. MR measurements were performed at room temperature (300 K). As the motor molecule, we chose a donor–acceptor substituted-type OCA^43^ to ensure visible light operation. The following measurements were performed with a 2’R-isomer of the OCA, unless otherwise noted. The OCA was placed in the device as its (2’R-M)-trans (hereinafter, M-trans) isomer or (2’R-P)-cis (hereinafter, P-cis) isomer. The results of spin-valve-like measurements displayed in Fig. 2b show a clear up-spin selectivity (the spin angular momentum is oriented parallel to the direction of electron propagation) for the M-trans device and down-spin selectivity (the spin angular momentum is oriented antiparallel to the direction of electron propagation) for the P-cis device, whose directions are consistent with those reported in previous CISS experiments^14,23,27,38^. Meanwhile, in the racemic compound, no MR signal was found (Fig. 2c), elucidating that the MR signals had originated from the SP induced by the molecular chirality of the OCA layers. These results clearly indicate the ability of OCA chirality to select the spin orientation. However, the SP could not be inverted by light irradiation, as shown in the same figure. This is attributable to the gold electrode that is macroscopically flexible in its thin (20 nm) form but is extremely rigid at the molecular length scale to allow for molecular motor rotation. Therefore, we decided to employ another type of electrode that exhibits flexibility at the nanoscale.

### MR measurements with PEDOT/PSS top electrodes

**Fig. 3:**
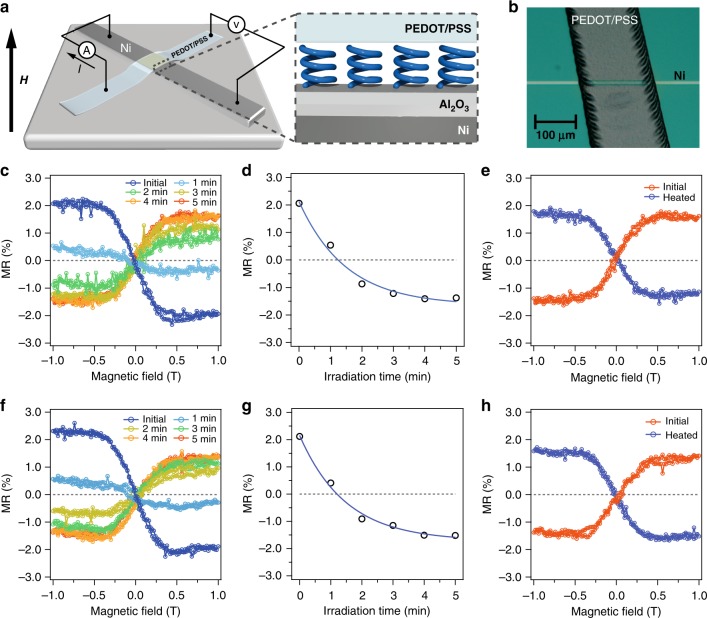
MR measurements with PEDOT/PSS top electrodes. **a** Schematics of the Ni(50 nm)-Al_2_O_3_(3 nm)-OCAs(2–3 nm)-PEDOT/PSS(600 nm) cross-bar tunnel junction device. Blue helices represents the OCAs (the actual molecular orientation might be disordered). **b** Top-view optical microscope image for the cross-bar device. **c** MR curves for the devices fabricated with M-cis isomer after various times of irradiation with visible light. **d** MR versus irradiation time for devices fabricated with M-cis isomer. Solid line denotes exponential fit. **e** MR curves for devices with P-trans isomer before and after thermal treatment at 80 °C for 12 h. **f** MR curves for devices fabricated with M-trans isomer after various times of irradiation with visible light. **g** MR versus irradiation time for devices fabricated with M-trans isomer. Solid line denotes exponential fit. **h** MR curves for devices with P-cis isomer before and after thermal treatment at 80 °C for 12 h. To evaluate the correlation between SP and chirality inversion correctly, a freshly fabricated new device was used in the experiment for the M-trans isomer (**f**, **g**, and **h**). Changes in MR curves during the molecular rotation of OCA in the same sample are shown in Supplementary Fig. 5

Hence, we fabricated a tunnel junction device by replacing gold with a poly(3,4-ethylenedioxythiophene):poly(styrenesulfonate) (PEDOT/PSS) (600 nm) electrode (Fig. 3a, b). First, we investigated the CISS effects with MR measurements to reproduce the result of devices with gold electrodes. Subsequently, we investigated the effects of photoirradiation on the CISS effects with this device. The MR curves were recorded after various visible light-irradiation times for a device fabricated with M-cis isomer, as shown in Fig. 3c. In the initial state, a clear antisymmetric MR curve with a negative slope was observed (blue curve). The irradiation time dependence of MR signals at −1 T are plotted in Fig. 3d. The MR signal decreased exponentially as light irradiation proceeded, and finally the sign of the MR signal was inverted to negative, indicating a light-induced spin switching in the spin-polarized current from up-spin selective to down-spin selective through the M-cis-to-P-trans photoisomerization of the OCA-film. A subsequent thermal activation process at 80 °C for the P-trans isomer inverted the signs of MR at −1 T from negative to positive again, as shown in Fig. 3e, implying a thermal-activation-induced spin switching from down-spin selective to up-spin selective through the P-trans-to-M-trans chirality inversion. Similar phenomena were observed in the device fabricated with the M-trans isomer, as shown in Fig. 3f (M-trans-to-P-cis photoisomerization), g (time dependence of MR), and h (P-cis-to-M-cis thermoisomerization). The phenomena with opposite sign were also observed in the device fabricated with 2’S isomers (Supplementary Fig. 3). This series of experiments clearly demonstrated that 4 times spin switching were induced during the 360° molecular rotation of the OCA. When compared with the device with gold electrode, the origin of the successful SP switching by the photoisomerization in the PEDOT/PSS device should be the nanoscale steric freedom in between the PEDOT polymer and inorganic Al_2_O_3_/Ni electrodes. Hence, the utilization of at least one flexible electrode is a crucial condition for the device fabrication. This result suggests the importance of flexibility at the molecular scale^44^ when molecular machines are integrated in solid-state devices.

### Spin-polarized conductive AFM measurements

**Fig. 4:**
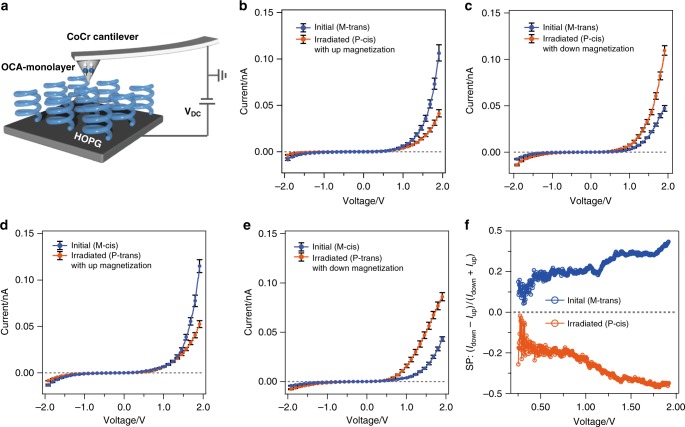
Spin-polarized conductive AFM measurements. **a** Schematic representation of spin-polarized conductive AFM measurements performed using ferromagnetic CoCr cantilever. Blue helices represents the OCAs (the actual molecular orientation might be disordered). **b** I–V curves measured with tip magnetized with up magnetic field orientation for M-trans isomer before and after irradiation with visible light. **c** I–V curves measured with tip magnetized with down magnetic field orientation for the M-trans isomer before and after irradiation with visible light. **d** I–V curves measured with tip magnetized with up magnetic field orientation for M-cis isomer before and after irradiation with visible light. **e** I–V curves measured with tip magnetized with down magnetic field orientation for the M-cis isomer before and after irradiation with visible light. **f** Spin polarization as a function of applied bias for M-trans isomer before and after irradiation with visible light. Error bars indicate standard errors

The absolute MR value of ~2% observed at each state is insignificant compared with other CISS experiments^23,35,37,38^. This is likely owing to the unpolarized leakage currents through the pinholes in the OCA films because the OCAs are not perfectly packed in the films. For an accurate estimation of the SP rate, we investigated a microscopic experiment, i.e., spin-polarized conductive AFM utilizing ferromagnetic tips. In these measurements, thin-films of OCAs were spin coated on freshly cleaved highly oriented pyrolytic graphite (HOPG) substrates. Current–voltage (*I*–*V*) measurements were performed in the contact mode with ferromagnetic CoCr tips (coercivity: 300 Oe) magnetized along the up or down direction (Fig. 4a). At least 40 *I*–*V* traces were recorded at different positions and subsequently averaged for each magnetized direction. The absolute current is always larger under a positive bias irrespective of molecular chirality. This asymmetric feature can be explained by the work function difference between the HOPG substrate and CoCr tip. The work functions of HOPG (in air) and CoCr tip are ~4.85 eV and 4.5 eV, respectively. This implies that the electron injection barrier is much lower at the tip–molecule interface than that at the substrate–molecule interface. Fig. 4b shows the averaged *I*–*V* traces for the device fabricated with the M-trans isomer measured with the up-direction magnetized CoCr tips before and after visible-light irradiation (the nonaveraged raw data are shown in Supplementary Fig. 6). The current flowing through the M-trans isomer (initial state) was higher compared with that through the P-cis isomer (after photoirradiation). Meanwhile, for CoCr tips that are magnetized in the down direction, the current flowing through the M-trans isomer (initial state) was smaller compared with that through the P-cis isomer (Fig. 4c), indicating light-induced spin switching in the spin-polarized current. Furthermore, we performed experiments for two other case scenarios: SP switching by photoisomerization from M-cis to P-trans measured with CoCr tip magnetized with up and down directions (Fig. 4d, e), separately. In all cases, the observed SP were up-spin selective for M-helicity molecules and down-spin selective for P-helicity molecules. This tendency is consistent with the results of MR measurements above. The SP rate in the M-trans isomer was calculated using the following formula:

1 $${\mathrm{SP}}\left( \% \right) = \left( {I_{{\mathrm{down}}} - I_{{\mathrm{up}}}} \right)/(I_{{\mathrm{down}}} + I_{{\mathrm{up}}}) \times 100$$

where *I*_up_ and *I*_down_ represent the currents when the magnetized direction of the CoCr tip is pointing up and down, respectively. The SP rate estimated at each bias is plotted in Fig. 4f. The SP rate enhanced gradually with increasing bias and finally reached 43% at +1.9 V. Meanwhile, for the photoirradiated states (P-cis isomer), the SP rate was estimated as –44% at +1.9 V. The magnitude of the SP rate in the present experiments is comparable with those in previous CISS experiments. However, the present experiments demonstrated the light-induced inversion of the SP direction in the spin-polarized currents, for the first time.

## Discussion

We now discuss the possible mechanism of the CISS effects in the present system. Hitherto, several theoretical mechanisms for the CISS effects have been proposed^45–49^. For instance, Matiyahu et al. recently proposed a theoretical model for CISS effects that is a simple tight binding model for electron transport through a single helical molecule, with SOIs on the bonds along the helix^48^. They introduced quantum interference through additional electron hopping between neighboring chains and allowed for the leakage of electrons from the helix to the environment to break a time-reversal symmetry. Consequently, a net spin polarization of the outgoing electrons was obtained. These results enabled a qualitative understanding of the mechanism for the CISS effects while the significant magnitude of SP rate (e.g., maximum of 43% in the current experiments) arising from small SOIs of organic molecules is still being investigated.

In this study, we performed the calculations for SOIs for the OCAs in one-electron reduced states by the contracted spin–orbit-configuration interaction (SO-CI) method. The electronic states (ϕ_*n*_; *n* is the index number) for the anion radical of M-trans were represented by singly excited configurations. The SOI was evaluated by < ϕ_*n*_ |*H*_SOI_|ϕ_*m*_ > where *H*_SOI_ is the SOI Hamiltonian, and subsequently diagonalized to obtain the spin–orbit states. This method has been employed for calculating intersystem crossing energies in a photoreactive molecule to yield reasonable values^50^. For the anion radical state, *n* and *m* were chosen within the LUMO to LUMO+25 levels (LUMO = lowest unoccupied molecular orbital for the neutral molecule). We have calculated these transition energies because the OCA molecule should be in a one-electron reduced state during the electron transmission process and should experience interstate transitions that start from a state in an electrode on one side and ends at a state in an electrode in the opposite side. Interestingly, we found that some matrix elements of the *n*–*m* interaction are extremely large, although most of the interactions are relatively weak (Supplementary Table 1 and Supplementary Fig. 8). Large values exceeding 30 cm^−1^ are shown for the transition between state 22 (LUMO+21) and state 25 (LUMO+24), for example (Supplementary Fig. 9). In these cases, the transitions contain the π-electron-to-lone-pair or π-electron-to-σ-electron excitation within the same sulfur atom, which should be associated with large orbital angular momentum changes in sulfur atomic orbitals. This result suggests the importance of SOIs within degenerate atomic orbitals in the CISS effect, which is reminiscent of strong SOIs in corrugated graphene and carbon nanotubes^51,52^. It is notable that the energy difference between up-spin and down-spin becomes twice as large as the SOI energy. Because the room temperature energy is ~200 cm^−1^, the SOI splitting of 60–70 cm^−1^ is more than one-third of the room temperature energy, which appears sufficient to explain the large SP observed in our experiments. Although we do not know why or how these strong SOIs affect the whole transmission process, the energy order appears reasonable despite the OCA being composed of only light elements. It is noteworthy that we did not use gold electrodes in the MR measurements (Fig. 3) that excludes the effect of heavy element electrodes that are often reported in literature.

In summary, we have performed MR and spin-polarized conductive AFM measurements that exhibit SP direction switching upon photoirradiation or heat treatment, by utilizing an artificial molecular motor. In these devices, the P/M chirality, which is the origin of SP generation through the CISS effect, is reconfigurable by external stimuli and thus represents a new type of organic spintronics device. During the development of this device, we found that the flexibility of the electrode at the nanoscale is important. The present results are beneficial for the development of next-generation organic photo/thermospintronic devices combined with molecular machines.

## Methods

### Synthesis and characterization of OCAs

OCAs were synthesized according to the reported procedure^43^. The cis and trans isomers were separated by silica gel column chromatography (n-hexane:CH_2_Cl_2_ = 2:1). The optical resolution was performed by a CHIRAL ART Cellulose-SB (SMC) column using n-hexane: methyl *tert*-butyl ether (65:35). The elution times were 2’S-P-trans: 10.5 min; 2’R-P-trans: 11.5 min; 2’S-M-trans: 12.5 min; 2’R-M-trans: 13.5 min for trans isomer, and 2’S-P-cis: 10.5 min; 2’R-P-cis: 11 min; 2’S-M-cis: 11.5 min; 2’R-M-cis: 13 min for cis isomer. The enantiopurity in each state confirmed by HPLC is >95%. CD spectra were recorded on J-720WI (JASCO) in toluene solution (Supplementary Fig.  1).

### Fabrication process of MR devices

The devices for the MR measurements were fabricated as follows: a Ni layer (50 nm) that acts as a bottom magnetic electrode was fabricated on a cleaned SiO_2_ substrate by optical lithography and sputtering. Subsequently, the Ni electrode was covered by an insulating Al_2_O_3_ layer (3 nm) by the sputtering method. The OCA layer was deposited by spin coating from toluene solution (2500 rpm, 30 s), followed by annealing at 50 °C for 1 h to evaporate the solvent. To prepare the PEDOT/PSS electrodes, we slightly modified a reported method^53^. A freestanding PEDOT/PSS film obtained in water was laminated once on a Teflon sheet to be dried. Subsequently, the PEDOT/PSS film was peeled off gently from the sheet using a pair of tweezers and laminated on the SiO_2_/Ni/Al_2_O_3_/OCA substrates with the crossbar configuration (Fig. 1). Finally, electrical contacts were established using silver paste.

### MR measurements

Electrical contacts were established in a standard four-probe configuration with a cross-bridge geometry. MR measurements were performed under reduced He pressure in a physical property measurement system (PPMS) (Quantum Design). A constant DC current of 1 μA was applied using the 2636B source meter (Keithley) and the potential difference across the junction was measured using the 2182 A nanovoltmeter (Keithley). A magnetic field of upto 1.0 T was applied perpendicular to the device interface. The temperature of the sample was controlled by the PPMS.

### Spin-polarized conductive AFM measurements

Spin-polarized conductive AFM measurements (*I–V* measurements) were performed using the JSPM-4210 scanning probe microscope (JEOL). In this experiment, OCA films were deposited on freshly cleaved HOPG substrates by spin coating from toluene solution (2500 rpm, 30 s), followed by annealing at 50 °C for 1 h to evaporate the solvent. The *I–V* measurements were recorded in the contact mode by applying the bias voltage to the tip (cantilever). CoCr tips (MESP-V2, Bruker) that were magnetized in the up and down magnetic field orientations were used for the *I–V* measurements. At least 40 *I–V* traces were recorded and averaged for each chiral state or magnetic field orientation. For each *I–V* measurement, the tip was placed in a new position.

### Irradiation process

Visible light was irradiated from the topside of the devices with a xenon light source (MAX-303: ASAHI SPECTRA). The desired wavelength of 436 ± 10 nm was selected using optical band-pass filters. The light intensity was ~10 mW/cm^2^. For the MR measurements, the light was guided into the PPMS through a quartz optical fiber of diameter 1.2 mm. All the physical measurements were performed after the irradiation was stopped. Light absorption by the PEDOT/PSS electrode can be ignored because the thin PEDOT–PSS film is almost transparent at λ = 436 nm.

### Computational details

The ground-state geometry of (2’R)-(M)-trans was optimized by DFT calculations with B3LYP^54^/6–31 G(*d*). To examine the SOIs in the reduced states, we performed contracted SO-CI^55,56^ calculations with the 6–31 G(*d*) plus diffuse *p* and *d* functions on N, O, and S atoms. The SO-CI includes singly excited-state configurations where an electron is placed at the LUMO to LUMO+25 in each reduced state. The one-electron operator was utilized in the SOI calculations. Gaussian 16 suite of programs^57^ were used for the DFT calculations. The SO-CI calculations were performed using the GAMESS 2013 program^58^.

## Supplementary information

Peer Review

Supplementary Information

## Data Availability

All data are available from the corresponding author upon reasonable request.

## References

[1] Rocha AR (2005). Towards molecular spintronics. Nat. Mater..

[2] Shim JH (2008). Large spin diffusion length in an amorphous organic semiconductor. Phys. Rev. Lett..

[3] Dediu VA, Hueso LE, Bergenti I, Taliani C (2009). Spin routes in organic semiconductors. Nat. Mater..

[4] Watanabe S (2014). Polaron spin current transport in organic semiconductors. Nat. Phys..

[5] Liu H (2018). Organic-based magnon spintronics. Nat. Mater..

[6] Cinchetti M, Dediu VA, Hueso LE (2017). Activating the molecular spinterface. Nat. Mater..

[7] Sun X (2017). A molecular spin-photovoltaic device. Science.

[8] Valenzuela SO, Tinkham M (2006). Direct electronic measurement of the spin Hall effect. Nature.

[9] Ishizaka K (2011). Giant Rashba-type spin splitting in bulk BiTeI. Nat. Mater..

[10] Jungwirth T, Wunderlich J, Olejnik K (2012). Spin Hall effect devices. Nat. Mater..

[11] Sanchez JC (2013). Spin-to-charge conversion using Rashba coupling at the interface between non-magnetic materials. Nat. Commun..

[12] Manchon A, Koo HC, Nitta J, Frolov SM, Duine RA (2015). New perspectives for Rashba spin-orbit coupling. Nat. Mater..

[13] Sinova J, Valenzuela SO, Wunderlich J, Back CH, Jungwirth T (2015). Spin Hall effects. Rev. Mod. Phys..

[14] Gohler B (2011). Spin selectivity in electron transmission through self-assembled monolayers of double-stranded DNA. Science.

[15] Naaman R, Waldeck DH (2012). Chiral-induced spin selectivity effect. J. Phys. Chem. Lett..

[16] Naaman R, Waldeck DH (2015). Spintronics and chirality: spin selectivity in electron transport through chiral molecules. Annu. Rev. Phys. Chem..

[17] Michaeli K, Varade V, Naaman R, Waldeck DH (2017). A new approach towards spintronics-spintronics with no magnets. J. Phys. Condens. Matter.

[18] Kettner M (2018). Chirality-dependent electron spin filtering by molecular monolayers of helicenes. J. Phys. Chem. Lett..

[19] Senthil Kumar K, Kantor-Uriel N, Mathew SP, Guliamov R, Naaman R (2013). A device for measuring spin selectivity in electron transfer. Phys. Chem. Chem. Phys..

[20] Dor OB, Morali N, Yochelis S, Baczewski LT, Paltiel Y (2014). Local light-induced magnetization using nanodots and chiral molecules. Nano Lett..

[21] Eckshtain-Levi M (2016). Cold denaturation induces inversion of dipole and spin transfer in chiral peptide monolayers. Nat. Commun..

[22] Xie Z (2011). Spin specific electron conduction through DNA oligomers. Nano Lett..

[23] Kiran V (2016). Helicenes--a new class of organic spin filter. Adv. Mater..

[24] Tassinari F (2018). Chirality dependent charge transfer rate in oligopeptides. Adv. Mater..

[25] Aragones AC (2017). Measuring the spin-polarization power of a single chiral molecule. Small.

[26] Dor OB, Yochelis S, Ohldag H, Paltiel Y (2018). Optical chiral induced spin selectivity XMCD study. CHIMA.

[27] Koplovitz G (2019). Single domain 10 nm ferromagnetism imprinted on superparamagnetic nanoparticles using chiral molecules. Small.

[28] Al-Bustami H (2018). Single nanoparticle magnetic spin memristor. Small.

[29] Ben Dor O, Yochelis S, Mathew SP, Naaman R, Paltiel Y (2013). A chiral-based magnetic memory device without a permanent magnet. Nat. Commun..

[30] Kumar A, Capua E, Fontanesi C, Carmieli R, Naaman R (2018). Injection of spin-polarized electrons into a AlGaN/GaN device from an electrochemical cell: evidence for an extremely long spin lifetime. ACS Nano.

[31] Mtangi W, Kiran V, Fontanesi C, Naaman R (2015). Role of the electron spin polarization in water splitting. J. Phys. Chem. Lett..

[32] Mtangi W (2017). Control of electrons’ spin eliminates hydrogen peroxide formation during water splitting. J. Am. Chem. Soc..

[33] Rosenberg RA, Mishra D, Naaman R (2015). Chiral selective chemistry induced by natural selection of spin-polarized electrons. Angew. Chem. Int. Ed. Engl..

[34] Banerjee-Ghosh K (2018). Separation of enantiomers by their enantiospecific interaction with achiral magnetic substrates. Science.

[35] Bloom BP, Kiran V, Varade V, Naaman R, Waldeck DH (2016). Spin selective charge transport through cysteine capped CdSe quantum dots. Nano Lett..

[36] Bloom BP, Graff BM, Ghosh S, Beratan DN, Waldeck DH (2017). Chirality control of electron transfer in quantum dot assemblies. J. Am. Chem. Soc..

[37] Koplovitz G (2017). Magnetic nanoplatelet-based spin memory device operating at ambient temperatures. Adv. Mater..

[38] Varade V (2018). Bacteriorhodopsin based non-magnetic spin filters for biomolecular spintronics. Phys. Chem. Chem. Phys..

[39] Koumura N, Zijlstra RW, van Delden RA, Harada N, Feringa BL (1999). Light-driven monodirectional molecular rotor. Nature.

[40] Feringa BL, van Delden RA, Koumura N, Geertsema EM (2000). Chiroptical molecular switches. Chem. Rev..

[41] Feringa BL (2001). In control of motion: from molecular switches to molecular motors. Acc. Chem. Res..

[42] van Leeuwen T, Lubbe AS, Štacko P, Wezenberg SJ, Feringa BL (2017). Dynamic control of function by light-driven molecular motors. Nat. Rev. Chem..

[43] van Delden RA, Koumura N, Schoevaars A, Meetsma A, Feringa BL (2003). A donor-acceptor substituted molecular motor: unidirectional rotation driven by visible light. Org. Bio. Chem..

[44] Eelkema R (2006). Molecular machines: nanomotor rotates microscale objects. Nature.

[45] Yeganeh S, Ratner MA, Medina E, Mujica V (2009). Chiral electron transport: scattering through helical potentials. J. Chem. Phys..

[46] Gutierrez R, Diaz E, Naaman R, Cuniberti G (2012). Spin-selective transport through helical molecular systems. Phys. Rev. B.

[47] Medina E, Gonzalez-Arraga LA, Finkelstein-Shapiro D, Berche B, Mujica V (2015). Continuum model for chiral induced spin selectivity in helical molecules. J. Chem. Phys..

[48] Matityahu S, Utsumi Y, Aharony A, Entin-Wohlman O, Balseiro CA (2016). Spin-dependent transport through a chiral molecule in the presence of spin-orbit interaction and nonunitary effects. Phys. Rev. B.

[49] Pan TR, Guo AM, Sun QF (2016). Spin-polarized electron transport through helicene molecular junctions. Phys. Rev. B.

[50] Kinoshita SN (2018). Different photoisomerization routes found in the structural isomers of hydroxy methylcinnamate. Phys. Chem. Chem. Phys..

[51] Huertas-Hernando D, Guinea F, Brataas A (2006). Spin-orbit coupling in curved graphene, fullerenes, nanotubes, and nanotube caps. Phys. Rev. B.

[52] Nakamura M, Castro EV, Dora B (2009). Valley symmetry breaking in bilayer graphene: a test of the minimal model. Phys. Rev. Lett..

[53] Greco F (2011). Ultra-thin conductive free-standing PEDOT/PSS nanofilms. Soft Matter.

[54] Becke AD (1993). Density-functional thermochemistry 3. The role of exact exchange. J. Chem. Phys..

[55] Koseki S, Schmidt MW, Gordon MS (1992). MCSCF/6-31G(d, p) calculations of one-electron spin-orbit-coupling constants in diatomic-molecules. J. Phys. Chem..

[56] Koseki S, Gordon MS, Schmidt MW, Matsunaga N (1995). Main-group effective nuclear charges for spin-orbit calculations. J. Phys. Chem..

[57] Frisch, M. J. et al. *Gaussian 16, Rev. B.01* (Gaussion Inc., Wallingford CT, 2016).

[58] Schmidt MW (1993). General atomic and molecular electronic-structure system. J. Comput. Chem..

