# Supplementary material for: Light-driven molecular switch for reconfigurable spin filters
Source: Nat Commun. 2019 Jun 5;10:2455. doi: 10.1038/s41467-019-10423-6 (PMC6549145; doi:10.1038/s41467-019-10423-6)
Supplement: Supplementary file 2 — Supplementary Information [file 41467_2019_10423_MOESM2_ESM.pdf]

Supplementary Information

Light-driven Molecular Switch for Reconfigurable Spin Filters

Suda et al.

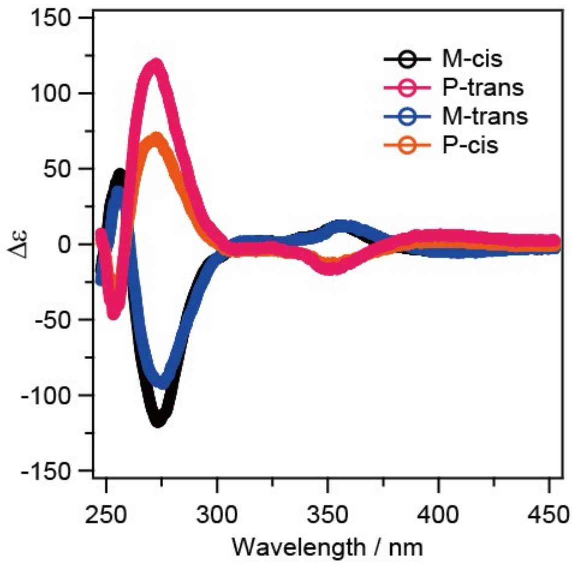

Supplementary Figure 1. CD spectra for the OCAs in toluene solution. Irradiation of the M-trans isomer with visible light (436 nm) induces a tran-to-cis photoisomerization resulting in P-cis isomer with opposite helicity. Subsequent thermal activation process at 50 °C invert the helicity to M again, by producing an M-cis isomer. The second photoisomerization processes proceed similarly, resulting in P helicity with P-cis isomer. Starting from the enantiopure M-cis isomer, the enantiopurity can be estimated for ~90 % for P-trans, ~70 % for M-trans, and ~60 % for P-cis isomer.

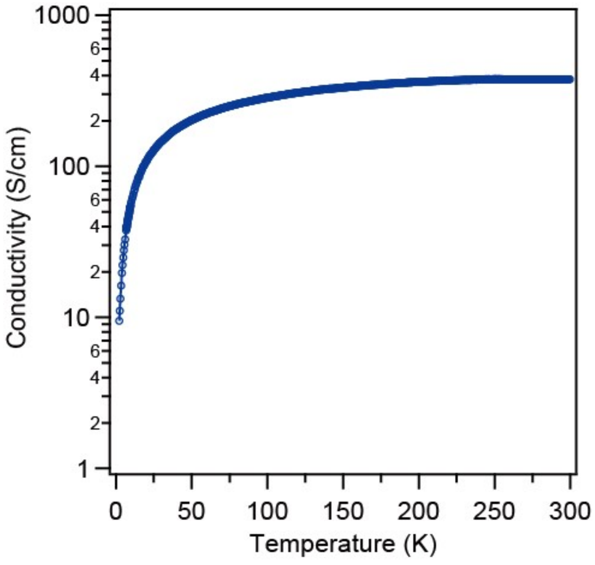

Supplementary Figure 2. Conductivity of PEDOT-PSS electrode.

a

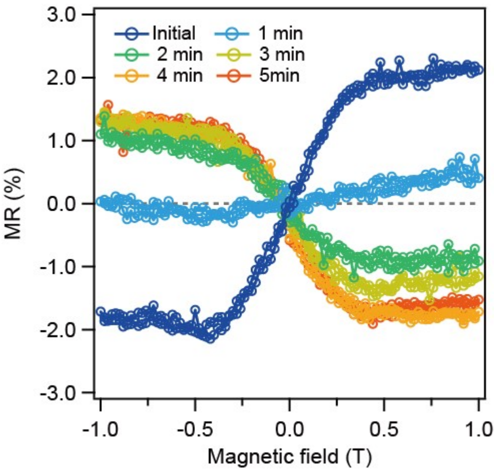

b

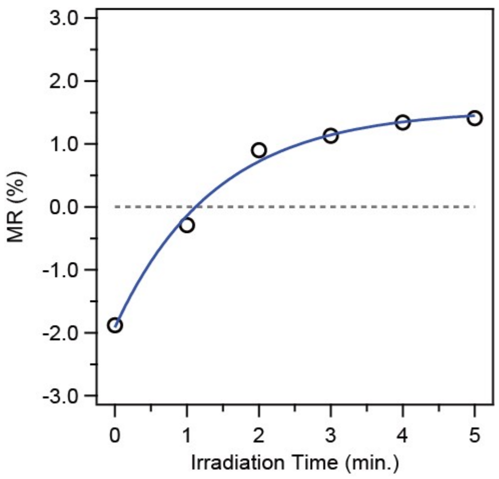

c

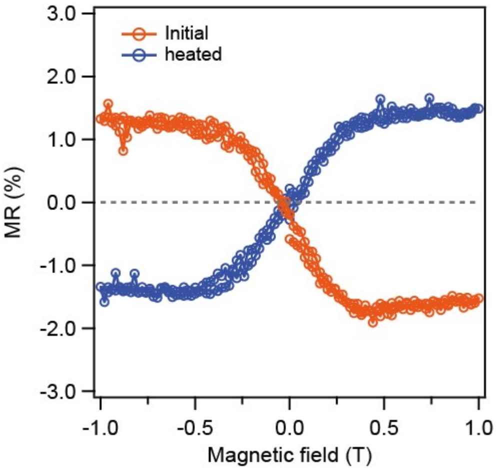

Supplementary Figure 3. Effects of photoirradiation and thermal treatments on MR curves for 2'S-isomers. a MR curves for devices fabricated with M-cis isomer after various times of irradiation with visible light. **b** MR versus irradiation time for devices fabricated with M-cis isomer. Solid line denotes exponential fit. **c** MR curves for devices with P-trans isomer before and after thermal treatment at 80 ℃.

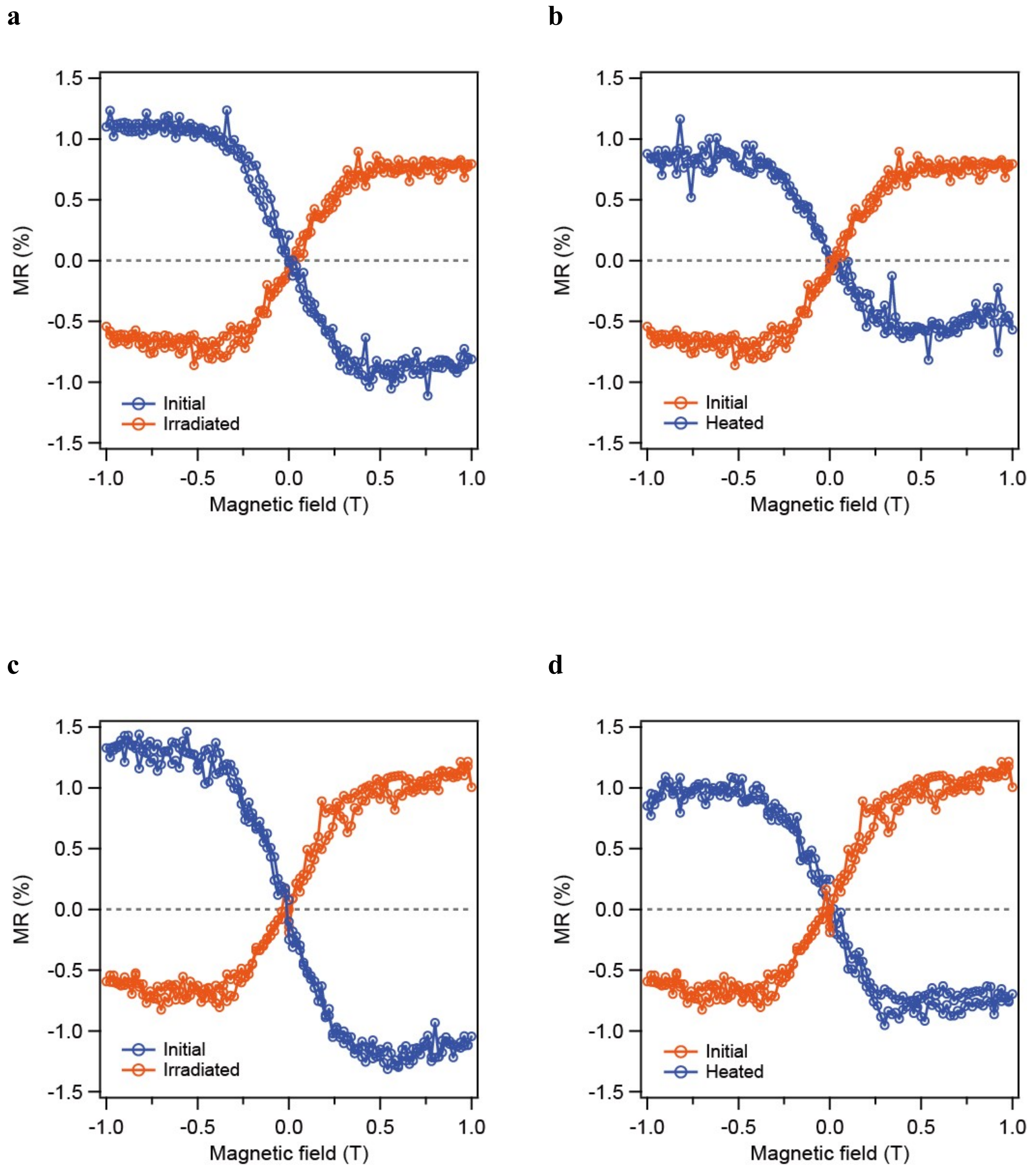

Supplementary Figure 4. Effects of photoirradiation and thermal treatments on MR curves for another device (2'R-isomers). a MR curves for the devices fabricated with M-cis isomer before and after irradiation with visible light. **b** MR curves for devices with P-trans isomer before and after thermal treatment at 80 ℃ for 12 h. **c**MR curves for devices fabricated with M-trans isomer before and after irradiation with visible light. **d** MR curves for devices with P-cis isomer before and after thermal treatment at 80 ℃ for 12 h. To evaluate the correlation between SP and chirality inversion correctly, a freshly fabricated new device was used in the experiment for the M-trans isomer (**c** and **d**).

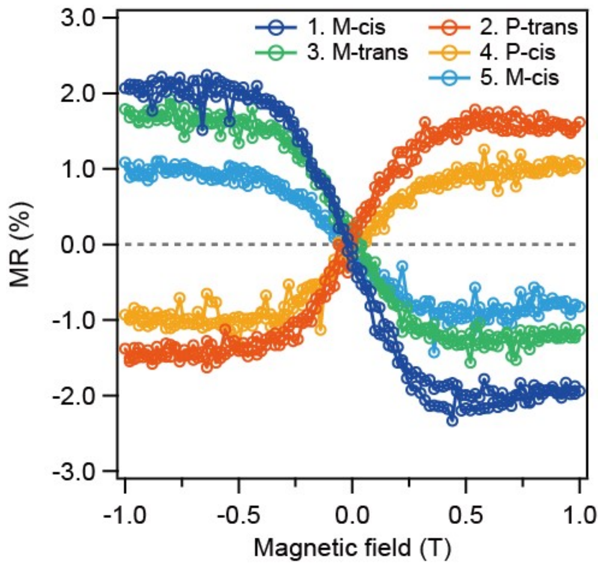

Supplementary Figure 5. Changes in MR curves during the molecular rotation of OCA in the same sample. Visible light irradiation of device with M-cis isomer induces a cis-to-trans photoisomerization, resulting in P-trans isomer with opposite helicity. Subsequent thermal activation process at 80 ℃ invert the helicity to M-trans again. The second photoisomerization processes proceed similarly, resulting in M helicity with M-trans isomer. Finally, second thermal activation process at 80 ℃ results in M-cis isomer again. Starting from the M-cis isomer with an MR of 2%, the MR value attenuated gradually to ~1% during 360° molecular rotation.

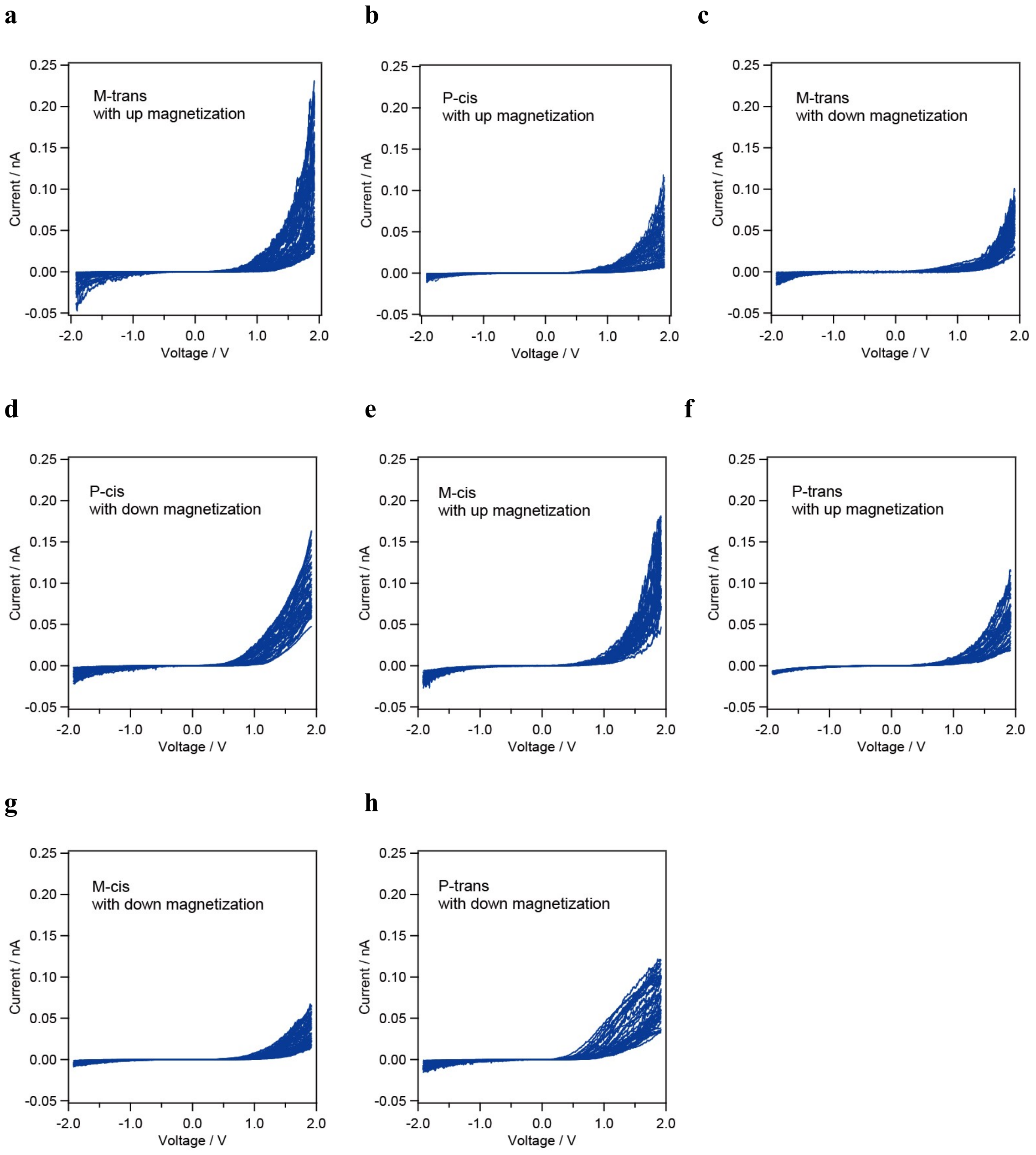

Supplementary Figure 6. I–V curves (nonaveraged raw data) for spin-polarized conductive AFM measurements. At least 40 I–V curves were recorded with tip magnetized with up or down magnetic field orientation by changing the position of the tip on each sample. The figures correspond to **a** M-trans with up magnetization, **b** P-cis with up magnetization, **c** M-trans with down magnetization, **d** P-cis with down magnetization, **e** M-cis with up magnetization, **f** P-trans with up magnetization, **g** M-cis with down magnetization, and **h** P-trans with down magnetization.

a **b**

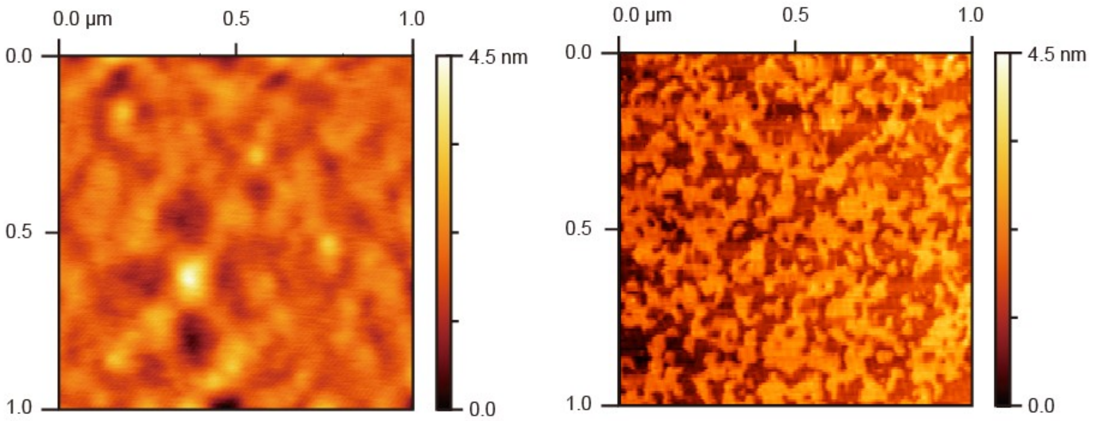

Supplementary Figure 7. AFM images for the OCA films. a Relatively thick film used for MR measurements that was spin coated from 1-mM toluene solution. **b** Relatively thin film used for spin-polarized conductive measurements that was spin coated from 0.25-mM toluene solution.

Supplementary Table 1. SOI energy for several transitions. Combinations of n and m are selected for high interaction energy values exceeding 20 cm^{-1}.

State		SOI energy (cm^{-1})
n	m	
22	25	34.64
23	25	33.22
11	24	23.93
23	24	23.79

Supplementary Table 2. Energies of SOMO with respect to HOMO for each anion radical state.

State	ΔE (cm^{-1})	State	ΔE (cm^{-1})	State	ΔE (cm^{-1})
1	68388	11	89480	21	104316
2	75411	12	92421	22	105940
3	79318	13	92640	23	107257
4	83093	14	98127	24	109211
5	84147	15	99356	25	112020
6	85178	16	99620	26	113073
7	85705	17	100717		
8	86056	18	101002		
9	87790	19	101375		
10	88097	20	102736		

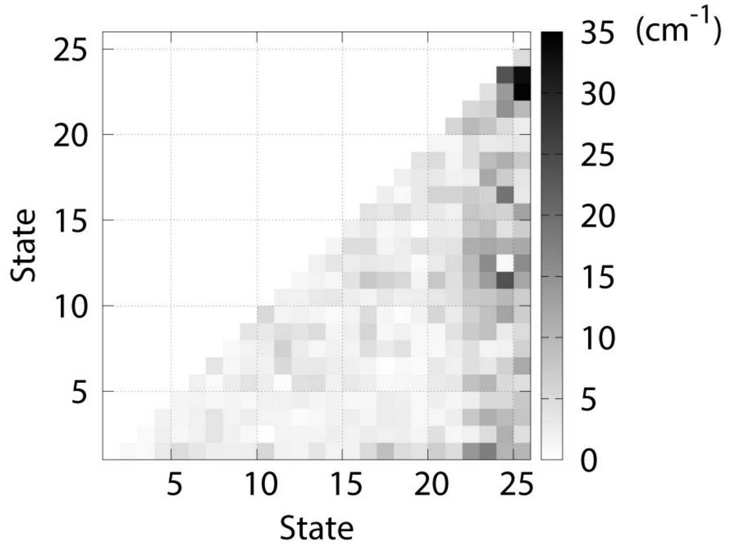

Supplementary Figure 8. SOI matrix for all interstate transitions. The interaction strength is expressed in grayscale (right bar) for each combination of start state (vertical axis) and final state (horizontal axis).

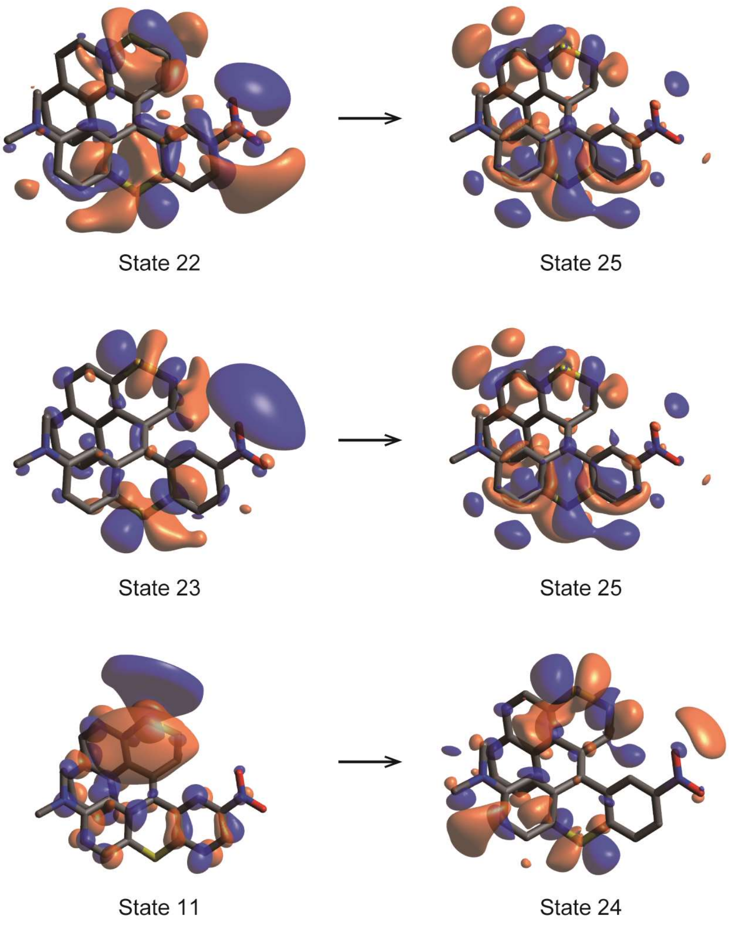

Supplementary Figure 9. Molecular orbitals for each anion radical state. Arrows represent interstate transitions with relatively large SOIs (see Table S1).
