# Supplementary material for: Light-driven molecular switch for reconfigurable spin filters
Source: Nat Commun. 2019 Jun 5;10:2455. doi: 10.1038/s41467-019-10423-6 (PMC6549145; doi:10.1038/s41467-019-10423-6)
Supplement: Supplementary file 1 — Peer Review [file 41467_2019_10423_MOESM1_ESM.pdf]

Reviewers' comments:

Reviewer #1 (Remarks to the Author):

The manuscript describes original and very significant work. It demonstrates for the first time the ability to switch spin selectivity by changing the handedness of chiral molecules, either by light or thermally. The authors also found that the "stiffness level" of the top electrode plays an important role in enabling the light induced conformation change. Because of its important, the authors should provide all the experimental details that will allow others to repeat or to follow this work. Some of this information is missing.
Specifically they have to address the following issues:
1. Light- The wavelength that was used, the power of the laser beam per area, the exact configuration of the illumination. How the light was introduced to the sample ? from the top through the top electrode? These are very important issues that have to be addressed in the main text.
2. How many devices were measured in each case? Experimental results should come with error estimation and in the graph one should have error-bars.
3. In the supplementary material the authors should ass details on the characterization of the molecules and specifically on the enantio-purity.
The manuscript should be published in Nat. Comm. but only after the authors respond to the above points.
Ron Naaman

Reviewer #2 (Remarks to the Author):

One of the most prominent challenges in the field of molecular spintronics at the moment is to exploit the unique properties of molecular components, such as their reactiveness to external stimuli, to generate second-generation spintronics devices with additional functionalities.
The manuscript of Suda and co-workers reports about a very significant progress towards this very ambitious goal. In particular, the authors show that they are able to invert the sign of the magneto-resistance in an organic spin-valve structure by irradiating the device using light. The sign of the MR is inverted because light irradiation induces a change in the chirality in the used molecular component, which in turn affects the sign of the spin polarization of the electrons that travel through the chiral molecule. Interestingly, the authors find that in order to obtain the switching, the Au electrode used in a first series of devices, needs to be replaced by a more flexible PEDOT/PSS electrode.

Due to the significant advancement in the field of molecular spintronics that is reported in this article, I think that it should be published in Nature Communications. The results and the data analysis are sound and I could not find any problem in the argumentation.
I have only some minor points:

1) First of all, I would like to suggest the authors to include the two following references. The first one is a Review article in Nature materials, that underlines the importance of generating functional molecular spintronics devices using the response of molecular systems to an external stimulus (Nature Materials 16, 507–515 (2017)). The second is one of the first reports of an optically-active spin-photovoltaic device (Science 357, 677–680 (2017)). I think that those two papers are extremely relevant to the topic of the manuscript and, as such, should be added in the references.

2) Second, at page 5, when the PEDOT/PSS electrode is introduced for the first time, the authors should explain what PEDOT/PSS actually is for the sake of clarity.

3) Third, I would suggest the authors to have a native English speaker to proof read the manuscript, in order to improve a little bit its readability.

Reviewer #3 (Remarks to the Author):

Report on Manuscript no. 201146 “Light-driven Molecular Switch for Reconfigurable Spin Filters” by Suda et al.

In the present manuscript, Suda et al. show the capability of chiral molecules to selectively transmit electrons of a preferred spin direction, an effect previously established as chirality-induced spin selectivity (CISS). A reversal of the molecular handedness reverses the preferred spin direction. Relying on light-induced inversion of the molecular chirality, the authors demonstrate for the first time a spin-filtering device that can be reconfigured by external stimuli.

For the studies of spin selectivity in electron transport layers of chiral, sterically overcrowded alkenes (OCAs) were adsorbed on a thin ferromagnetic nickel layer. A gold layer, and alternatively a PEDOT/PSS layer was deposited on top of the molecular layer to contact the far side of the molecules. By magnetizing the nickel layer in up or down direction, the electron injection into the molecular layer was polarized accordingly. The spin polarization obtained from the CISS effect in the layer was measured as the magnetoresistance of the device, i.e., the change of the electric resistance induced by the respective substrate magnetization, normalized to the resistance measured on an unmagnetized substrate.

Irradiation with light at 435nm is expected to induce a cis-trans isomerization, which corresponds to a twist around the central C=C double bond and is accompanied by switching of the molecular chirality. Starting from an M-trans isomer, the process is expected to yield the P-cis isomer. Both isomers were found to exhibit opposite MR characteristics, i.e., preferential transmission of electrons with opposite spin orientations. In the first gold-coated device, however, no reversal of the magnetoresistance was observed after irradiation. The authors hypothesize that the molecular rotation is hindered by the gold coating, which might be too rigid on a molecular length scale. Indeed, a light-induced reversal of the spin selectivity was observed in a revised device, in which the gold layer was replaced by a more flexible conductive polymer film. Subsequent to the isomerization, a thermal activation step (annealing) allows to invert the molecular chirality by changing the enantiomer as which the molecules are present on the surface. Combining two irradiation and two annealing steps, a complete unidirectional rotation of the upper part of the molecule can be achieved. The cycle is manifested in four distinct inversions of the MR characteristics.

However, the absolute MR values of about 2% are low compared to the asymmetries reported elsewhere. To the reviewer’s knowledge, no data on the CISS effect in motor molecules are available yet. For hepta-helicene as a comparably small chiral organic molecule spin polarization values of P = 6% to 8% were reported by Kettner et al. (ref. 16). The authors assume that leak holes in the OCA layer give rise to unpolarized currents that reduce the measured selectivity.

To address this issue, the spin selectivity was investigated more locally by recording I-V curves with a magnetized AFM tip. These measurements yielded asymmetries of over 40%, which is a huge effect but comparable to earlier studies at other systems. The sign of selectivity is consistent with the MR results.

The present work is first to demonstrate not only a reduction but a reversal of the preferred spin orientation upon an external stimulus such as irradiation with light. The findings can hence be considered highly relevant and are likely to inspire further research, an achievement that should warrant publication in Nature Communications which I strongly support.

Before publication the authors are requested to address the following comments.

Comments:
- How are the molecules oriented in the solid state structures (fig. 2 and fig. 3)? Are the molecules lying “flat”, with the thioxanthene and the naphto-thiopyran moieties touching the same Al2O3 or gold surfaces (except being helically twisted) or are they standing upright between both electrodes with the xantene moiety touching the aluminumoxide and the naphto-pyran the gold or PEDOT/PSS electrode ? Figure 1 with the schematic spirals and spin directions suggest the latter, but I guess the former is more likely. A statement in the text should clarify this.

- Related to this question: when the molecules are lying flat, do you ensure that only the xanthene moiety is in contact with the aluminumoxide ? Or, do you have an idea about the distribution between molecules where the xanthene and those where the nafto-pyran touches the aluminumoxide ? Or is that distribution a random one ?

- It would be instructive to determine the racemization barrier in the solid system.

- Do the molecules show a long-range lateral order ?

- In figures 2 and 3 the authors show a thin layer between the aluminumoxide and the molecules without denoting its nature.

- In fig. 3 the irradiation times for the switching of the MR curves are given. This irradiation time certainly depends on the irradiation fluence. Please state the fluence applied to the samples at the wavelength employed.

- In fig. S1 CD spectra of the enantiomers are shown. Are they taken in solution or in the solid state ?

- The calculation yields S-O interactions as shown in Table S1 and fig. S5. It is astonishing that this interaction is relatively weak for most of the states, but for state numbers greater than 21 this interaction becomes much stronger. What is the reason for that behavior ?

- Connected to the above remark, what are the energies of the states 5 to 25, either relative to the HOMO or relative to the vacuum level ?

- From the AFM measurements, the selectivity was calculated as the difference between the currents for both magnetization directions of the tip; in the MR measurements, the normalized difference between electric resistances for the respective magnetization and an unmagnetized substrate was used instead. The comparability of both methods could be improved if the authors consistently took either the difference between both magnetization directions or referred to the unmagnetized substrate or tip in both cases.

- The authors assign the low MR values to pinholes in the OCA layers. It should be elucidated further whether these holes are a consequence of the packing of the molecules on the surface or due to general issues related to the layer quality, that is, irregularly and randomly distributed defects. It should also be discussed if the SP is reduced due to the general layer quality, due to oppositely oriented molecules upon deposition (see comment above), or due to an incomplete switching process in the layer.

- Only little information is given about the temporal behavior and the repeatability of the chirality inversion processes. In figure 3 (c), the initial M-cis molecule exhibits stronger MR than the P-trans form, which is obtained after 5 min irradiation. In figure 3 (e), the MR is larger for the initial P-trans form than for M-cis obtained after heating. At least for M-cis and P-trans, this does not correspond to the relative CD intensities in figure S1. Does further irradiation or further heating increase the magnitude of MR to the level of the initial signal? The data could indicate that the MR and thus the chirality is reduced during each chirality inversion step. How large is the MR after several full rotation

cycles? As the time scales of both the light-induced and thermal chirality switching processes (and their reduction) might be relevant for future applications, this issue could be discussed further.

- In the AFM I-V curves, the absolute current is always larger if a positive voltage is applied, no matter which molecular chirality is present. In contrast, one might expect point-symmetrical curves. Therefore, the issue should briefly be discussed.

Minor Remarks:
Page 5, line 17: Thermal activation temperature is stated as 80° C for P-trans → M-trans. In Figure S1, 50° C is stated for P-cis → M-cis. Is this correct, and what gives rise to the energy difference?
In the last sentence of the caption, "P-cis" has to be changed to "P-trans".

Page 11, figure 1: The isomerization steps are indicated as horizontal arrows. What do you want to express by the small additional arrows, pointing in opposite direction ? Usually, a different wavelength is required for the reverse switching.

Page 18, line 4: The evolution time is missing.
It would be helpful to highlight the MR = 0% line in all magnetoresistance plots (and I = 0 nA in the I-V plots) to emphasize the respective symmetry.

It would be further helpful for a reader when for the different irradiation times (figs. 3 and S3) more distinct colors were used. The same holds for the I – V curves (fig. 4).

Response to Reviewer’s comments

<Original comments of Reviewer 1>

The manuscript describes original and very significant work. It demonstrates for the first time the ability to switch spin selectivity by changing the handedness of chiral molecules, either by light or thermally. The authors also found that the "stiffness level" of the top electrode plays an important role in enabling the light induced conformation change. Because of its important, the authors should provide all the experimental details that will allow others to repeat or to follow this work. Some of this information is missing.

Specifically they have to address the following issues:

1. Light- The wavelength that was used, the power of the laser beam per area, the exact configuration of the illumination. How the light was introduced to the sample ? from the top through the top electrode? These are very important issues that have to be addressed in the main text.
2. How many devices were measured in each case? Experimental results should come with error estimation and in the graph one should have error-bars.
3. In the supplementary material the authors should ass details on the characterization of the molecules and specifically on the enantio-purity.

The manuscript should be published in Nat. Comm. but only after the authors respond to the above points.

Ron Naaman

<The author’s point-by-point responses to Reviwer 1>

We appreciate your review for our manuscript. We have revised the introductory paragraph of our manuscript in accordance with your comments, by removing and adding a few descriptions. All changes to our manuscript are highlighted in yellow background in the manuscript files.

Comment 1

Light- The wavelength that was used, the power of the laser beam per area, the exact configuration of the illumination. How the light was introduced to the sample ? from the top through the top electrode? These are very important issues that have to be addressed in the main text.

Reply 1

We added the description for the detailed irradiation process in the "Methods" section as follows:

“5. Irradiation process. Visible light was irradiated from the topside of the devices with a xenon light source (MAX-303: ASAHI SPECTRA). The desired wavelength of 436 ± 10 nm was selected using optical band-pass filters. The light intensity was ~10 mW/cm^2. For the MR measurements, the light was guided into the PPMS through a quartz optical fiber of diameter 1.2 mm. All the physical measurements were performed after the irradiation was stopped. Light absorption by the PEDOT/PSS electrode can be ignored because the thin PEDOT-PSS film is almost transparent at λ = 436 nm.”

Comment 2

How many devices were measured in each case? Experimental results should come with error estimation and in the graph one should have error-bars.

Reply 2

For the MR measurements, at least two samples were measured in each case. The absolute MR values are highly sample dependent, probably owing to the random pinhole distribution in the OCA films because the OCAs are not perfectly packed in the films, as mentioned in the manuscript. Hence, the use of error bars is not suitable for the MR data. Instead, we added the results from another device to the supplementary information in Fig. S4.

For spin-polarized conductive AFM measurements, we measured only one sample for each state. However, at least 40 *I*–*V* traces were recorded at different positions and they were averaged for each chiral state or magnetic field orientation. For each I–V measurement, the tip was placed in a new position. Error bars (standard errors) are added in Figs. 4b–e. Furthermore, all the raw data are shown in Fig. S6.

Comment 3

In the supplementary material the authors should ass details on the characterization of the molecules and specifically on the enantio-purity.

Reply 3

Immediately after the optical separation, the enantiopurity in each state confirmed by HPLC is > 95%. These highly pure enantiomers were used for the device fabrication. We added the description regarding the enantiopurity in the “Methods” section as follows.

“The enantiopurity in each state confirmed by HPLC is > 95%.”

At the device interfaces, the enantiopurity for each state was difficult to estimate because we could not measure the CD or NMR spectra in the solid state at the present stage. Meanwhile, the enantiopurity for each state in the solution phase can be estimated by analyzing the CD spectra. Starting from the pure M-cis isomer, the enantiopurity can be estimated for 90% for P-trans, ~70% for M-trans, and ~60% for P-cis isomer. We added the description regarding the enantiopurity in solution phase to the caption of Fig. S1 as follows:

“Starting from the enantiopure M-cis isomer, the enantiopurity can be estimated for ~90% for P-trans, ~70% for M-trans, and ~60% for P-cis isomer.”

<Original comments of Reviewer 2>

One of the most prominent challenges in the field of molecular spintronics at the moment is to exploit the unique properties of molecular components, such as their reactiveness to external stimuli, to generate second-generation spintronics devices with additional functionalities.
The manuscript of Suda and co-workers reports about a very significant progress towards this very ambitious goal. In particular, the authors show that they are able to invert the sign of the magneto-resistance in an organic spin-valve structure by irradiating the device using light. The sign of the MR is inverted because light irradiation induces a change in the chirality in the used molecular component, which in turn affects the sign of the spin polarization of the electrons that travel through the chiral molecule. Interestingly, the authors find that in order to obtain the switching, the Au electrode used in a first series of devices, needs to be replaced by a more flexible PEDOT/PSS electrode.

Due to the significant advancement in the field of molecular spintronics that is reported in this article, I think that it should be published in Nature Communications. The results and the data analysis are sound and I could not find any problem in the argumentation.
I have only some minor points:

1) First of all, I would like to suggest the authors to include the two following references. The first one is a Review article in Nature materials, that underlines the importance of generating functional molecular spintronics devices using the response of molecular systems to an external stimulus (Nature Materials 16, 507–515 (2017)). The second is one of the first reports of an optically-active spin-photovoltaic device (Science 357, 677–680 (2017)). I think that those two papers are extremely relevant to the topic of the manuscript and, as such, should be added in the references.

2) Second, at page 5, when the PEDOT/PSS electrode is introduced for the first time, the authors should explain what PEDOT/PSS actually is for the sake of clarity.

3) Third, I would suggest the authors to have a native English speaker to proof read the manuscript, in order to improve a little bit its readability.

<The author’s point-by-point responses to Reviwer 2>

We appreciate your review for our manuscript. We have revised the introductory paragraph of our manuscript in accordance with your comments, by removing and adding a few descriptions. All changes to our manuscript are highlighted in yellow background in the manuscript files.

Comment 1

First of all, I would like to suggest the authors to include the two following references. The first one is a Review article in Nature materials, that underlines the importance of generating functional molecular spintronics devices using the response of molecular systems to an external stimulus (Nature Materials 16, 507–515 (2017)). The second is one of the first reports of an optically-active spin-photovoltaic device (Science 357, 677–680 (2017)). I think that those two papers are extremely relevant to the topic of the manuscript and, as such, should be added in the references.

Reply 1

We added these important papers to the reference as Ref. 6 and Ref. 7

Comment 2

Second, at page 5, when the PEDOT/PSS electrode is introduced for the first time, the authors should explain what PEDOT/PSS actually is for the sake of clarity.

Reply 2

We changed "PEDOT/PSS" to "poly(3,4-ethylenedioxythiophene):poly(styrenesulfonate) (PEDOT/PSS)."

Comment 3

Third, I would suggest the authors to have a native English speaker to proof read the manuscript, in order to improve a little bit its readability.

Reply 3

The revised manuscript was edited by professional native editors at Editage, a division of Cactus Communications, to ensure that the language is clear and free of errors.

<Original comments of Reviewer 3>

Report on Manuscript no. 201146 “Light-driven Molecular Switch for Reconfigurable Spin Filters” by Suda et al.

In the present manuscript, Suda et al. show the capability of chiral molecules to selectively transmit electrons of a preferred spin direction, an effect previously established as chirality-induced spin selectivity (CISS). A reversal of the molecular handedness reverses the preferred spin direction. Relying on light-induced inversion of the molecular chirality, the authors demonstrate for the first time a spin-filtering device that can be reconfigured by external stimuli.

For the studies of spin selectivity in electron transport layers of chiral, sterically overcrowded alkenes (OCAs) were adsorbed on a thin ferromagnetic nickel layer. A gold layer, and alternatively a PEDOT/PSS layer was deposited on top of the molecular layer to contact the far side of the molecules. By magnetizing the nickel layer in up or down direction, the electron injection into the molecular layer was polarized accordingly. The spin polarization obtained from the CISS effect in the layer was measured as the magnetoresistance of the device, i.e., the change of the electric resistance induced by the respective substrate magnetization, normalized to the resistance measured on an unmagnetized substrate.

Irradiation with light at 435nm is expected to induce a cis-trans isomerization, which corresponds to a twist around the central C=C double bond and is accompanied by switching of the molecular chirality. Starting from an M-trans isomer, the process is expected to yield the P-cis isomer. Both isomers were found to exhibit opposite MR characteristics, i.e., preferential transmission of electrons with opposite spin orientations. In the first gold-coated device, however, no reversal of the magnetoresistance was observed after irradiation. The authors hypothesize that the molecular rotation is hindered by the gold coating, which might be too rigid on a molecular length scale. Indeed, a light-induced reversal of the spin selectivity was observed in a revised device, in which the gold layer was replaced by a more flexible conductive polymer film. Subsequent to the isomerization, a thermal activation step (annealing) allows to invert the molecular chirality by changing the enantiomer as

which the molecules are present on the surface. Combining two irradiation and two annealing steps, a complete unidirectional rotation of the upper part of the molecule can be achieved. The cycle is manifested in four distinct inversions of the MR characteristics.

However, the absolute MR values of about 2% are low compared to the asymmetries reported elsewhere. To the reviewer’s knowledge, no data on the CISS effect in motor molecules are

available yet. For hepta-helicene as a comparably small chiral organic molecule spin polarization values of P = 6% to 8% were reported by Kettner et al. (ref. 16). The authors assume that leak holes in the OCA layer give rise to unpolarized currents that reduce the measured selectivity.

To address this issue, the spin selectivity was investigated more locally by recording I-V curves with a magnetized AFM tip. These measurements yielded asymmetries of over 40%, which is a huge effect but comparable to earlier studies at other systems. The sign of selectivity is consistent with the MR results.

The present work is first to demonstrate not only a reduction but a reversal of the preferred spin orientation upon an external stimulus such as irradiation with light. The findings can hence be considered highly relevant and are likely to inspire further research, an achievement that should warrant publication in Nature Communications which I strongly support.

Before publication the authors are requested to address the following comments.

Comments:
-How are the molecules oriented in the solid state structures (fig. 2 and fig. 3)? Are the molecules lying "flat", with the thioxanthene and the naphto-thiopyran moieties touching the same Al2O3 or gold surfaces (except being helically twisted) or are they standing upright between both electrodes with the xantene moiety touching the aluminumoxide and the naphto-pyran the gold or PEDOT/PSS electrode ? Figure 1 with the schematic spirals and spin directions suggest the latter, but I guess the former is more likely. A statement in the text should clarify this.

-Related to this question: when the molecules are lying flat, do you ensure that only the xanthene moiety is in contact with the aluminumoxide ? Or, do you have an idea about the distribution between molecules where the xanthene and those where the nafto-pyran touches the aluminumoxide ? Or is that distribution a random one ?

-It would be instructive to determine the racemization barrier in the solid system.

-Do the molecules show a long-range lateral order ?

-In figures 2 and 3 the authors show a thin layer between the aluminumoxide and the molecules without denoting its nature.

-In fig. 3 the irradiation times for the switching of the MR curves are given. This irradiation time certainly depends on the irradiation fluence. Please state the fluence applied to the samples at the wavelength employed.

-In fig. S1 CD spectra of the enantiomers are shown. Are they taken in solution or in the solid state ?

-The calculation yields S-O interactions as shown in Table S1 and fig. S5. It is astonishing that this interaction is relatively weak for most of the states, but for state numbers greater than 21 this interaction becomes much stronger. What is the reason for that behavior ?

-Connected to the above remark, what are the energies of the states 5 to 25, either relative to the HOMO or relative to the vacuum level ?

-From the AFM measurements, the selectivity was calculated as the difference between the currents for both magnetization directions of the tip; in the MR measurements, the normalized difference between electric resistances for the respective magnetization and an unmagnetized substrate was used instead. The comparability of both methods could be improved if the authors consistently took either the difference between both magnetization directions or referred to the unmagnetized substrate or tip in both cases.

-The authors assign the low MR values to pinholes in the OCA layers. It should be elucidated further whether these holes are a consequence of the packing of the molecules on the surface or due to general issues related to the layer quality, that is, irregularly and randomly distributed defects. It should also be discussed if the SP is reduced due to the general layer quality, due to oppositely oriented molecules upon deposition (see comment above), or due to an incomplete switching process in the layer.

-Only little information is given about the temporal behavior and the repeatability of the chirality inversion processes. In figure 3 (c), the initial M-cis molecule exhibits stronger MR than the P-trans form, which is obtained after 5 min irradiation. In figure 3 (e), the MR is larger for the initial P-trans form than for M-cis obtained after heating. At least for M-cis and P-trans, this does not correspond to the relative CD intensities in figure S1. Does further irradiation or further heating increase the magnitude of MR to the level of the initial signal? The data could indicate that the MR and thus the chirality is reduced during each chirality inversion step. How large is the MR after several full rotation cycles? As the time scales of both the light-induced and thermal chirality switching processes (and their reduction) might be relevant for future applications, this issue could be

discussed further.

-In the AFM I-V curves, the absolute current is always larger if a positive voltage is applied, no matter which molecular chirality is present. In contrast, one might expect point-symmetrical curves. Therefore, the issue should briefly be discussed.

Minor Remarks:

Page 5, line 17:Thermal activation temperature is stated as 80° C for P-trans → M-trans. In Figure S1, 50° C is stated for P-cis → M-cis. Is this correct, and what gives rise to the energy difference?

In the last sentence of the caption, “P-cis” has to be changed to “P-trans”.

Page 11, figure 1:The isomerization steps are indicated as horizontal arrows. What do you want to express by the small additional arrows, pointing in opposite direction ? Usually, a different wavelength is required for the reverse switching.

Page 18, line 4:The evolution time is missing.

It would be helpful to highlight the MR = 0% line in all magnetoresistance plots (and I = 0 nA in the I-V plots) to emphasize the respective symmetry.

It would be further helpful for a reader when for the different irradiation times (figs. 3 and S3) more distinct colors were used. The same holds for the I – V curves (fig. 4).

<The author’s point-by-point responses to Reviwer 3>

We appreciate your review for our manuscript. We have revised the introductory paragraph of our manuscript in accordance with your comments, by removing and adding a few descriptions. All changes to our manuscript are highlighted in yellow background in the manuscript files.

Comment 1

How are the molecules oriented in the solid state structures (fig. 2 and fig. 3)? Are the molecules lying “flat”, with the thioxanthene and the naphto-thiopyran moieties touching the same Al2O3 or gold surfaces (except being helically twisted) or are they standing upright between both electrodes with the xantene moiety touching the aluminumoxide and the naphto-pyran the gold or PEDOT/PSS electrode ? Figure 1 with the schematic spirals and spin directions suggest the latter, but I guess the former is more likely. A statement in the text

should clarify this.

Related to this question: when the molecules are lying flat, do you ensure that only the xanthene moiety is in contact with the aluminumoxide ? Or, do you have an idea about the distribution between molecules where the xanthene and those where the nafto-pyran touches the aluminumoxide ? Or is that distribution a random one ?

Reply 1

We added the AFM images for the OCA films used in the spin-polarized conductive AFM measurements to the supplementary information as Fig. S7b (For the MR measurements, a relatively thicker film was used to reduce the pinholes). The molecular island shows a uniform thickness with a height of ~1.5 nm, indicating the monolayer formation of the OCA on the HOPG surface. However, the molecular arrangement in the monolayer is unclear at the present stage. The molecules may exhibit random orientations.

However, we would like to emphasize that finite spin-polarization owing to CISS effects can be expected even for a completely random orientation because the molecular chirality cannot be cancelled out. Currently, we are attempting to fabricate highly oriented OCA films with a designed anchoring group in which a higher spin-polarization can be expected. For a clearer understanding, we added the description regarding the random molecular orientation to the main text as follows.

"The OCA film was prepared by the spin-coating method and a random molecular arrangement is expected (AFM images for the OCA films are shown in Fig. S7)."

Comment 2

It would be instructive to determine the racemization barrier in the solid system.

Reply 2

Because we cannot measure the NMR or CD spectrum in the solid-state, it is impossible to estimate the racemization barrier in the solid state at the present stage. The racemization barrier of the OCA in solution was already determined by NMR measurements for 92.7 kJ/mol (ref. 43). The thermal activation temperature is 50 °C in solution and 80 °C in the solid films. This is reasonable when one considers the higher steric hindrance in the solid state compared to the solution phase. Hence, at the present stage, we can conclude that the racemization barrier in the solid state is higher than 92.7 kJ/mol.

Comment 3

Do the molecules show a long-range lateral order ?

Reply 3

As also mentioned in Reply 1, the OCA probably exhibits a random orientation in the film and hence the molecules have no long-range lateral order.

Comment 4

In figures 2 and 3 the authors show a thin layer between the aluminumoxide and the molecules without denoting its nature.

Reply 4

As also mentioned in Reply 1, we added the AFM images of the OCA film fabricated on Al_2O_3 to the supplementary information as Fig. S7a. The thickness is not uniform but is between 2–3 nm, and is sufficiently thin to unsure the electron tunneling process.

Comment 5

In fig. 3 the irradiation times for the switching of the MR curves are given. This irradiation time certainly depends on the irradiation fluence. Please state the fluence applied to the samples at the wavelength employed.

Reply 5

As also mentioned for the answer to Q1 by reviewer 1, the light intensity is ~10 mW/cm^2 at λ = 436 nm. We added the illumination details in the "Methods" section.

Comment 6

In fig. S1 CD spectra of the enantiomers are shown. Are they taken in solution or in the solid state ?

Reply 6

The CD spectra were obtained in toluene solution. We changed the caption of Fig. S1 from "CD spectra for the OCAs" to "CD spectra for the OCAs in toluene solution."

Comment 7

The calculation yields S-O interactions as shown in Table S1 and fig. S5. It is astonishing that this interaction is relatively weak for most of the states, but for state numbers greater than 21 this interaction becomes much stronger. What is the reason for that behavior ?

Reply 7

This point is relevant for understanding the origin of the spin current. We analyzed the details of all the transitions. The transitions with large SOI (which is proportional to $\Delta l \bullet \Delta s$, where Δl and Δs denote the orbital and spin angular momentum changes during the transition, respectively,) contain the π-*n** or *n*-π* excitation within the same sulfur atom, which should be associated with large orbital angular momentum changes (Δl) of sulfur atomic orbitals, while others do not exhibit such changes in large amplitudes in sulfur. Thus, we believe that the origin of the strong S–O interaction originates from the large orbital angular momentum changes in sulfur. This statement has been already described in p. 9.

Comment 8

Connected to the above remark, what are the energies of the states 5 to 25, either relative to the HOMO or relative to the vacuum level ?

Reply 8

This issue is also relevant for the characterization of the relevant transitions. We listed the energies of the SOMO with respect to the HOMO for each anion radical state in Table S2. These energies indicate a continuous energy spectrum for the anion radical states. No distinct energy difference is found between the transitions that indicate small SOIs and those indicating large SOIs.

Comment 9

From the AFM measurements, the selectivity was calculated as the difference between the currents for both magnetization directions of the tip; in the MR measurements, the normalized difference between electric resistances for the respective magnetization and an unmagnetized substrate was used instead. The comparability of both methods could be improved if the authors consistently took either the difference between both magnetization directions or referred to the unmagnetized substrate or tip in both cases.

Reply 9

As mentioned by the reviewer, in the MR measurements, the percentage of MR was calculated from the in-field-measured resistance, $R(H)$, and the zero-field resistance $R(H = 0)$ as MR(%) = 100 × [R (H) − R $(H = 0)$]/ R $(H = 0)$). However, we can expand this formula to the difference between the resistance for both magnetization directions, as follows: MR (up - down) = 100 × [R (H = 10000 Oe) − R (H = -10000 Oe)]/ R (H = 0). Hence, the comparability of both methods is possible only from the data shown in the manuscript.

In the spin-polarized conductive AFM measurements, the tips were magnetized using a strong permanent magnet before the measurements. A complete demagnetization of the tip is difficult at the present stage.

Comment 10

The authors assign the low MR values to pinholes in the OCA layers. It should be elucidated further whether these holes are a consequence of the packing of the molecules on the surface or due to general issues related to the layer quality, that is, irregularly and randomly distributed defects. It should also be discussed if the SP is reduced due to the general layer quality, due to oppositely oriented molecules upon deposition (see comment above), or due to an incomplete switching process in the layer.

Reply 10

We believe the low MR values are primarily owing to the "pinholes" and not significantly dependent on the "random orientation" or "incomplete switching process" of the OCA film.

First, in the spin-polarized conductive AFM measurements (implies microscopic measurements to avoid the pinholes), we measured for at least 40 times and the tip was placed in a new position for every measurement. For most of the measurements, we observed the spin-polarization tendency. Furthermore, only when the measurements were performed on the pinholes the excessive current that was independent of the tip magnetization direction was observed. If random molecular orientation was the reason, we would have observed the strong position dependence of the current on the film. Hence, we can exclude the possibility of "random orientation."

Next, low MR values were always observed even in the devices freshly prepared from enantiopure molecules. Hence, we can exclude the possibility of "incomplete switching process."

In addition, a similar phenomenon (low MR values owing to the pinholes) was reported in a previous report (ref. 23).

Comment 11

Only little information is given about the temporal behavior and the repeatability of the chirality inversion processes. In figure 3 (c), the initial M-cis molecule exhibits stronger MR than the P-trans form, which is obtained after 5 min irradiation. In figure 3 (e), the MR is larger for the initial P-trans form than for M-cis obtained after heating. At least for M-cis and P-trans, this does not correspond to the relative CD intensities in figure S1. Does further irradiation or further heating increase the magnitude of MR to the level of the initial signal? The data could indicate that the MR and thus the chirality is reduced during each chirality inversion step. How large is the MR after several full rotation cycles? As the time scales of both the light-induced and thermal chirality switching processes (and their reduction) might be relevant for future applications, this issue could be discussed further.

Reply 11

As mentioned in Reply 3 for Reviewer 1, photo- and thermal-induced racemization proceeded gradually during the molecular rotation. Hence, the MR signal was attenuated gradually by repeating the process. To evaluate the correlation between spin polarization and chirality inversion correctly, in the MR measurements, we prepared two types of freshly fabricated devices: one is with M-cis and the other with M-trans. Hence, Figures 3c, d, and e were based on the first sample, while Figures 3f, g, and h were based on the second sample. After a 5-min irradiation, both devices reached the photostationary state. In the thermal treatment, stationary states were obtained after 12 h. For a clearer understanding, we added the description regarding this to the caption of Figure 3 as follows.

"To evaluate the correlation between spin polarization and chirality inversion correctly, a freshly fabricated new device was used in the experiment for M-trans isomer (**f, g** and **h**). Changes in MR curves during the molecular rotation of OCA in the same sample are shown in Figure S5."

Furthermore, we added the MR signal change during molecular rotation in the same device to the supplementary information as Fig. S4.

Comment 12

In the AFM I-V curves, the absolute current is always larger if a positive voltage is applied, no matter which molecular chirality is present. In contrast, one might expect point-symmetrical curves. Therefore, the issue should briefly be discussed.

Reply 12

As mentioned by the reviewer, the absolute current is always larger if a positive voltage is applied. This asymmetric feature can be explained by the work function difference between the HOPG substrate and CoCr tip. Literature values of the work function of HOPG (in air) and CoCr tip are reported as ~4.85 eV and 4.5 eV, respectively (our measurements were performed in air). This implies that the electron injection barrier is much lower for the tip–molecule interface than for the substrate–molecule interface. Hence, the absolute current is always larger if a positive voltage is applied. We added the description regarding this to the main text as follows:

"The absolute current is always larger under a positive bias irrespective of molecular chirality. This asymmetric feature can be explained by the work function difference between the HOPG substrate and CoCr tip. The work function of HOPG (in air) and CoCr tip are ~4.85 eV and 4.5 eV, respectively. This implies that the electron injection barrier is much lower for the tip–molecule interface than for the substrate–molecule interface."

Comment 13

Page 5, line 17:Thermal activation temperature is stated as 80° C for P-trans → M-trans. In Figure S1, 50° C is stated for P-cis → M-cis. Is this correct, and what gives rise to the energy difference?

Reply 13

The descriptions are correct. The thermal activation temperature is 50 °C in solution and 80 °C in the solid films. This is reasonable when one considers the higher steric hindrance in the solid state compared to the solution phase.

Comment 14

In the last sentence of the caption, "P-cis" has to be changed to "P-trans".

Reply 14

"P-cis" is correct. Figure 3e shows the thermal activation process for P-trans, and Figure 3h shows the thermal activation process for P-cis.

Comment 15

Page 11, figure 1:The isomerization steps are indicated as horizontal arrows. What do you want to express by the small additional arrows, pointing in opposite direction ? Usually, a different wavelength is required for the reverse switching.

Reply 15

We had intended to express the reverse isomerization process. However, as mentioned by the reviewer, only the wavelength of 436 nm was used in our measurement. The small arrows in Fig. 1 were deleted.

Comment 16

Page 18, line 4:The evolution time is missing.

Reply 16

This was our mistake. We have corrected the mistake in the revised manuscript.

Comment 17

It would be helpful to highlight the MR = 0% line in all magnetoresistance plots (and I = 0 nA in the I-V plots) to emphasize the respective symmetry.

Reply 17

We highlighted the MR = 0% and I = 0 A lines in all graphs.

Comment 18

It would be further helpful for a reader when for the different irradiation times (figs. 3 and S3) more distinct colors were used. The same holds for the I – V curves (fig. 4).

Reply 18

We changed the colors for all MR and I–V curves to more distinct colors.

REVIEWERS' COMMENTS:

Reviewer #1 (Remarks to the Author):

The authors responded properly to all the comments of all reviewers and in my opinion the manuscript should now be accepted to Nature Communication. It is really and very nice advancement in the field of molecular spintronics.

Reviewer #2 (Remarks to the Author):

I think that the reply of the authors to the main concerns of the referees is satisfactory and the manuscript should now be published in Nat are Communications.
Before publication, however, the authors should add a short comment in the caption of figure 2, where they explicitly say that the orientation of the molecules its not ordered as suggested by the schematic picture. They should also add a sentence explaining that chrarlity plays a role venue in the present case where the monolayer shows a random growth.
This is a very important piece of information that should be explicitly mentioned in the manuscript.

Reviewer #3 (Remarks to the Author):

The authors gave satisfying answers to the comments of the reviewers.
Therefore I suggest to accept the manuscript in the present form, and to publish it in Nature Communications.

Response to Reviewer's comments

<Original comments of Reviewer 2>

I think that the reply of the authors to the main concerns of the referees is satisfactory and the manuscript should now be published in Nat are Communications.

Before publication, however, the authors should add a short comment in the caption of figure 2, where they explicitly say that the orientation of the molecules its not ordered as suggested by the schematic picture. They should also add a sentence explaining that chrarlity plays a role venue in the present case where the monolayer shows a random growth.

This is a very important piece of information that should be explicitly mentioned in the manuscript.

<The author's point-by-point responses to Reviewer 2>

We appreciate your review for our manuscript. We have revised the our manuscript in accordance with your comments. We used the tracked changes feature to make these changes for our revised manuscript.

Comment 1

Before publication, however, the authors should add a short comment in the caption of figure 2, where they explicitly say that the orientation of the molecules its not ordered as suggested by the schematic picture.

Reply 1

We added a sentence " *the actual molecular orientation might be disordered* ." to the caption of Fig. 2a, 3a and 4a.

Comment 2

They should also add a sentence explaining that chrarlity plays a role venue in the present case where the monolayer shows a random growth.

Reply 2

We added a sentence "However, we emphasize that finite spin-polarization owing to CISS effects can be expected even for a completely random orientation because the molecular chirality cannot be cancelled out." to the main text (page4, line 10).
